# Movement examination of the lumbar spine using a developed wearable motion sensor

**DOI:** 10.1049/htl2.12063

**Published:** 2023-12-09

**Authors:** Reza Abbasi‐Kesbi, Mohammad Fathi, Seyed Zaniyar Sajadi

**Affiliations:** ^1^ MEMS & NEMS Department, Faculty of New Sciences and Technologies University of Tehran Tehran Iran; ^2^ Department of Biomedical Engineering, Faculty of Medical Sciences and Technologies Islamic Azad University Science and Research Branch Tehran Iran; ^3^ School of Electrical and Computer Engineering, College of Engineering University of Tehran Tehran Iran

**Keywords:** biomedical measurement, health care, patient monitoring

## Abstract

A system for monitoring spinal movements based on wearable motion sensors is proposed here. For this purpose, a hardware system is first developed that measures data of linear acceleration, angular velocity, and the magnetic field of the spine. Then, the obtained data from these sensors are combined in a proposed complementary filter, and their angular variations are estimated. The obtained results of angular variation of this system in comparison with an accurate reference illustrate that the root mean squared error is less than 1.61 degrees for three angles of $\phi_{r}$, $\theta_{r}$ and $\psi_{r}$ for this system that proves this system can accurately estimate the angular variation of the spine. Then, the system is mounted on the lumbar spine of several volunteers, and the obtained angles from the patients' spine are compared with some healthy volunteers' spine, and the performance of their spine improves over time. The results show that this system can be very effective for patients who suffer from back problems and help in their recovery process a lot.

## Graphical abstract

A wearable system for monitoring spinal movements is proposed here. At first, the angular variation of the lumbar spine of some patients is estimated by a presented complementary filter and the developed wearable system. Then, the estimated angular variation is compared with the spinal angle of some healthy persons. The obtained results of the developed system shows the performance of patients who have serious problems in their lumar spine improve over time after manual therapy and some repetitive tests that is presented in the paper.

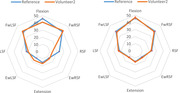

## 1 INTRODUCTION

Intervertebral disc resorption is one of the most common causes of low back pain [1]. Disc failure is not a disease but a natural process that occurs with age in all people who undergo degeneration [2]. Degeneration literally means aging. This is due to excessive disk work. Repeated blows to the disc over a lifetime will gradually wear it out and make it old and damaged. However, the pain of some people develops faster and at a younger age, and in others later. Also, some blows and injuries to the spine can accelerate this process [3]. Some strenuous physical activity can put a lot of pressure on the disc and cause it to rupture prematurely. There is also a genetic predisposition in some families that causes the disc to break down at a younger age [4].

The process of decay and aging of the disc between the vertebrae takes place in an almost constant sequence [5]. Initially, the nucleus or central nucleus of the disc loses its ability to absorb water, and its water content decreases. The nucleus then thickens and becomes like a ring around itself, that is, the annulus [6]. Following these changes, the shock absorption ability, which is an important property of the intervertebral disc, decreases and the blows are applied directly to the vertebrae instead of being consumed in the disc [7]. This not only causes the vertebrae and joints between them to rupture and become arthritic sooner but also puts even more pressure on the disc. Gradually, small ruptures form in the annulus. These ruptures gradually become larger and larger, weakening the disc. Gradually the disk size decreases. Its height decreases and its elasticity decreases [8]. Decreasing the height of the disc causes the vertebrae to be closer to each other, and this proximity causes the joints between the vertebrae or the same joints to be more compressed, and the result of this compression is wear and tear [9]. Osteoarthritis causes pain in the joints between the vertebrae. Thus, it can be seen that the source of many spinal diseases that eventually lead to low back pain is intervertebral disc failure. Disc failure itself can cause pain and back pain [10].

Combined lumbar spine movement is recommended for clinical examination because it provides information about the mechanical patterns of pain. In one study, with the help of a computer and the necessary equipment, back pain was examined, and then with the help of manual therapies, the pain was somewhat reduced [11]. For this purpose, a volunteer was evaluated before manual treatments in the lumbar region and afterward with the help of a computer. Their results explain how computer‐aided reference and combined movement examination (CME) can be used objectively for diagnostic information and as a result in mechanical low back pain (LBP) cases [11]. In another study, 151 normal individuals aged 20 to 69 years were evaluated using a combined movement examination between the L1 and S1 levels of the spine [12]. Cases of sciatica and degenerative low back pain were evaluated before and after therapeutic interventions in a combined motor examination. Score changes were obtained to examine the combined motion and all outcome measures. Their results indicate the acceptable reliability of combined motor examination when recording lumbar motion in normal individuals. In their clinical cases, the limitations of lumbar rotation were consistent with their reported scores for pain and disability. The results showed significant improvement after all interventions, especially in the most limited direction of combined motion examination [12]. Another study was performed to evaluate the use of a computer‐assisted hybrid motion test to measure changes in lumbar movement after pain management intervention in 17 cases of lumbar spondylosis [13]. The results showed that seven of the 17 participants stated that a “combined” movement was the most painful direction for their CME. Additionally, four participants had a significant improvement, five of them experienced more than 30% improvements in low back function, and for eight of them, low back pain was more annoying than stiffness. Furthermore, six of them clinically had the least difference in self‐reported pain [13]. A subset of cases to specific groups of structure provides insight into the different patterns of CME motion. Thus, the data from their study, provide early evidence for intervertebral disc, maxillary joint, and CME motor patterns of root nerve compression in cases of chronic lumbar spondylosis [13].

Because there are various devices for measuring the position of the spine, a wide range of technologies are under these systems, the most common of which is the use of inertial units (IMU) [14, 15, 16]. For example in a study, office workers spend long hours sitting in front of computers, which inevitably leads to spinal problems [17]. Ergonomic strategies to maintain the correct posture, such as a standing desk, have to some extent helped to reduce occupational hazards in the posture position, but subconscious deviation from the intended correct posture is inevitable [17]. Wearable systems, which own the ability to monitor the condition of the spine and provide real‐time feedback alerts on the condition of the spine, allow workers to correct their condition and, therefore, reduce overall spinal weakness [18].

Here, a system lightweight and portable for the assessment of spinal diagnosis is proposed. For this purpose, a hardware system is first developed that can measure data of the spine by accelerometer, gyroscope, and magnetometer sensors. Then, with the help of a complementary filter, the angle variations of this spine are estimated. The obtained angular variation is compared to a reference, and the obtained results show that the root mean squared error of the angles is less than 1.61 degrees. By mounting the developed sensor on the spin lumbar of patients and performing some defined tests, some data is extracted. The obtained results of the lumbar spine from the patient are compared with some healthy persons as a reference value. Although there are meaningful differences between the plot at first, the amount of error declines over time. The results show that this system can be very effective for patients who suffer from spinal problems, special for in‐home or in a clinic.

## 2 MATERIAL AND METHOD

**FIGURE 1:**
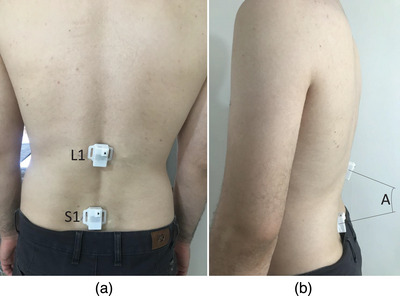
The sensor placement over L1 and S1 levels for measuring the angular variations.

The main purpose of the paper is to develop a low‐cost and wearable system for diagnosing primary spinal problems for patients and improving their spinal function. For this purpose, first, a system is developed that measures the angular movements of the lumbar spine. The developed sensor is placed on the volunteer's *L*_1_ and *S*_1_ levels (Figure 1a,b). In a relaxed standing position, participants have their lumbar lordosis (*A*) that is obtained by Equation (1), which is the difference between L1 and S1:

(1) $$A=|L_{1}-S_{1}|$$

where *S*_1_ and *L*_1_ are sacrum and lumbar level 1, respectively, that consist of ϕ, θ, and ψ defined as the angle around *x*, *y*, and *z* axes in turn. After mounting the developed system on the volunteers' lumbar spine, several special tests are considered, and then, the angular movements of these exercises are recorded and compared to the reference to make a point for the quality of the movement. The following equation illustrates the error between the angular variation of patients and the healthy person who is considered as a reference.

(2) $$\begin{array}{ccc} E_{A}\left(\%\right) & = & \frac{\sum_{i=1}^{n}\frac{|A_{e,i}-A_{r,i}|*100}{A_{r,i}}}{n} \end{array}$$

where $A_{e,i}$ is the estimated angular variation (lumbar lordosis) for the lumbar spine of patients while the $A_{r,i}$ are reference angles, respectively. Also, *n* is the number of tests that are defined in the result section and its value is eight. As Equation (2) shows, the estimated angles are subtracted from reference values. The obtained error demonstrates the amount of quality of movement. If the value is tended to 0, the movement is true; otherwise, the volunteers have to repeat the movement properly until the error is tended to 0. In order to estimate angular movement, data from the gyroscope, accelerometer, and compass sensors are fused in a complementary filter that results in obtaining the angular variations of the lumbar spine. First, the presented complementary filter is described and then how to develop the presented system is explained in comprehensive.

### 2.1 Angular variation estimation

Errors in motion sensors have adverse effects on the sensor output and eliminating them has always been a crucial issue in angular variation systems [19]. The errors are divided into stochastic and bias categories that the latter can be eliminated by calibration, while stochastic errors are random and are not removed by calibration [20]. In this situation, some filters should be used to get rid of the stochastic errors. In other words, the filters are employed to eliminate random errors and estimate the angular movements using the data from the accelerometer, gyroscope, and compass [21]. There are various methods for reducing the random errors such as the Kalman filter, extended Kalman filter, and complementary filter. Here, a complementary filter is proposed to estimate angular variation of ϕ, θ, and ψ with at least calculations according to the following equation:

(3) $$\begin{array}{ccc} \phi & = & tan^{-1}\left(\frac{q_{2}q_{3}-q_{0}q_{1}}{q_{0}^{2}+q_{3}^{2}-0.5}\right)\\ \theta & = & -sin^{-1}\left(2q_{1}q_{3}+2q_{0}q_{2}\right),\\ \psi & = & tan^{-1}\left(\frac{q_{1}q_{2}-q_{0}q_{3}}{q_{0}^{2}+q_{1}^{2}-0.5)}\right) \end{array}$$

where ϕ, θ, and ψ are angular variations around the *x*‐axis, *y*‐axis, and *z*‐axis. As Equation (2) shows, the angular variation works based on the quaternion data. Each quaternion, $q_{n,t}$, can contain the following components that are mentioned in Equation (3):

(4) $$\begin{array}{c} q_{n,t}=\left[q_{1,t},\;q_{2,t},\;q_{3,t},\;q_{4,t}\right]. \end{array}$$

The filter is based on quaternion data and its derivative.

(5) $${\dot{q}}_{n,t}={\dot{q}}_{g,t-1}-\alpha \left({\dot{q}}_{a,t-1}+{\dot{q}}_{m,t-1}\right),$$

in which ${\dot{q}}_{n,t}$ is the derivative of quaternion obtained from a combination of gyroscope, accelerometer, and magnetometer data. Also, ${\dot{q}}_{g,t-1}$, ${\dot{q}}_{a,t-1}$, and ${\dot{q}}_{m,t-1}$ are, respectively, derivatives of quaternion for gyroscope, accelerometer, and magnetometer data in the previous period. As Equation (4) shows, the accelerometer data is fused with magnetometer data and then multiplied by an attenuation factor (α). Indeed, only a three‐dimensional angle can be extracted with the help of an accelerometer. However, it should be noted that the angular variation obtained from accelerometer data is effective for a long time and is not valid in a short time. Therefore, numerous deviations over time emerge that cannot be an acceptable result for the analysis of angular movements [22]. By combining magnetometer data with an accelerometer, reliable data is obtained that can be very helpful in estimating the angle. As mentioned earlier, the gyroscope is also a motion sensor that measures the amount of angular velocity. Although a gyroscope has reliable data in the short term, in the long run, this data can be misleading. Nonetheless, the fusion of accelerometer and magnetometer data is subtracted from the gyroscope data in order to reduce this long‐term deviation of the gyroscope. It should be noted, however, that this operation is performed in a quaternion derivative that all of the descriptions are done in Equation (4). Finally, the quaternion value is extracted from Equation (5) with the help of simple integration:

(6) $$q_{n,t}=q_{n,t-1}+{\dot{q}}_{n,t}▵t,$$

where ${\dot{q}}_{n,t}$ and $q_{n,t}$ are derivatives of quaternion and quaternion in the current time while ${\dot{q}}_{n,t-1}$ is related to quaternion data in a previous time. Also, ▵t is the sample rate of sensor data and its value is 40 ms.

In order to obtain the quaternion derivative of the gyroscope, accelerometer, and magnetometer data, the following methods are defined. First, the data of the three‐axis gyroscope is converted into the quaternion derivative by Equation (6) [23].

(7) $${\dot{q}}_{g,t}=\frac{1}{2}q_{n,t-1}⊗\left(w_{r,t}-e_{bg}\right),$$

in which $w_{r,t}$ is the raw data of the gyroscope that consist of $w_{x}$, $w_{y}$ and $w_{z}$. Moreover, $e_{bg}$ is the bias of the gyroscope, and $q_{n,t-1}$ is the quaternion data in the previous time [24]. Accelerometer data also are converted to the quaternion derivative by Equation (7):

(8) $${\dot{q}}_{a,t}=\frac{da_{g,t}}{dq}a_{u,t},$$

where $a_{g,t}$ and $R_{t-1}$ are gravitational acceleration and rotational matrix, respectively, that are defined by Equations (8) and (9) [25]. Also, $a_{u,t}$ is new accelerometer data and is modelled based on subtracting the raw accelerometer data and gravitational acceleration (Equation 10):

(9) $$a_{g,t}=R_{r,t-1}\left[\begin{array}{c} 1\\ 0\\ 0 \end{array}\right]$$

(10) $$R_{r,t-1}=\left[\begin{array}{ccc} q_{1}^{2}+q_{2}^{2}-0.5 & \;q_{2}q_{3}+q_{1}q_{4} & \;q_{2}q_{4}-q_{1}q_{3}\\ q_{2}q_{3}-q_{1}q_{4} & \;q_{1}^{2}+q_{3}^{2}-0.5 & \;q_{3}q_{4}+q_{1}q_{2}\\ q_{2}q_{4}+q_{1}q_{3} & \;q_{3}q_{4}-q_{1}q_{2} & \;q_{1}^{2}+q_{4}^{2}-0.5 \end{array}\right]$$

(11) $$a_{u,t}=a_{r,t}-a_{g,t}$$

Magnetometer data also are converted to the quaternion derivative by following equations.

(12) $${\dot{q}}_{m,t}=\frac{ds_{i,t}}{dq}m_{u,t}$$

where $s_{i,t}$ and $m_{u,t}$ are soft iron effect and new magnetometer data in turn. Magnetometer data is modelled as follows:

(13) $$m_{u,t}=m_{r,t}-s_{i,t}-H_{i,t}$$

in which $H_{i,t}$, and $m_{r,t}$ are hard iron and raw magnetometer data, respectively. The hard iron effect is calculated by the summation of minimum and maximum raw magnetometer divided on 2 and $s_{i,t}$ is defined as follows [26]:

(14) $$s_{i,t}=R_{r,t-1}\left[\begin{array}{c} \sqrt{h_{x,t}^{2}+h_{y,t}^{2}}\\ 0\\ h_{z,t} \end{array}\right]$$

where *h* is the compass data obtained by subtracting the raw data from the hard iron and multiplying this value by the matrix *R* [26]:

(15) $$\left[\begin{array}{c} h_{x,t}\\ h_{y,t}\\ h_{z,t} \end{array}\right]=R_{r,t-1}\left[\begin{array}{c} m_{r,x,t}-H_{i,x,t}\\ m_{r,y,t}-H_{i,y,t}\\ m_{r,z,t}-H_{i,z,t} \end{array}\right]$$

## 3 HARDWARE

**FIGURE 2:**
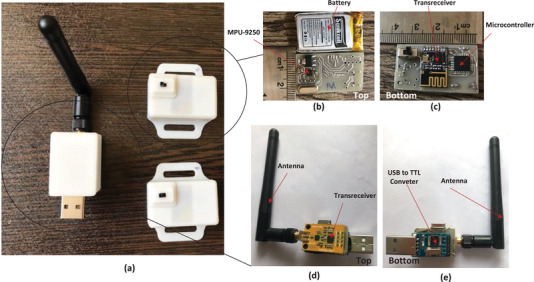
(a) The measurement setup, (b,c) sensory nodes and their components, and (d,e) the central node and their components.

The developed measurement setup is developed and shown in Figure 2a which consists of two sensor nodes and one central node. To implement the proposed system, first, the circuit schematics are designed, and then the printed circuit board is completed. Finally, the surface mount device (SMD) electrical components are soldered on the board, and a box is developed by 3D printing to prevent dust and other harmful components on the boards.

As Figure 2a demonstrates, the developed system consists of two sensory nodes and one collector (central node). Each sensory nodes include a motion sensor, a processor, a transceiver, and a battery (Figure 2b,c). The motion sensor is an MPU‐9250 that is a digital sensor whose output is $I_{2}C$ [27]. The transceiver also has an ISP output to communicate with the processor and also a planar inverted‐F antenna (PIFA) for sending data wirelessly. Thus, the required processor in the sensory nodes is selected to have both of these protocols (ISP, and $I_{2}C$) and that is an Atmega 8 AVR series microcontroller [28]. The motion sensor information is read via the microcontroller's $I_{2}C$ protocols and delivered to the transceiver via the ISP protocol [29]. The power consumption of the sensory nodes is very low, and it is supplied with the help of a lithium polymer battery of 3.7 V and 300 mAh for 10 h. This system can be charged after discharging the battery energy by a cell phone charger.

On the other side, the central node is developed which consists of three main parts: the processor, the transmitter, and a TTL to USB converter (Figure 1d,e). As mentioned earlier, the transceiver benefits from an ISP protocol, and to communicate with such a transceiver, it should be selected a processor with this advantage. In addition, the converter works based on the UART protocol, and the selected process should be owned by the protocols. Also, the data is received wirelessly by an antenna, as can be seen in Figure 1d,e. If the volunteers have more distance between the sensor node and the central node, the large antenna can see data without any limitations while using PIFA makes some limitations of the distance between the sensor and central node. The program written on this microcontroller first receives the received data from the transceiver and then sends them to the TTL to USB converter to send the data to a personal computer and then save and further processing.

## 4 RESULTS

### 4.1 System accuracy measurement

**FIGURE 3:**
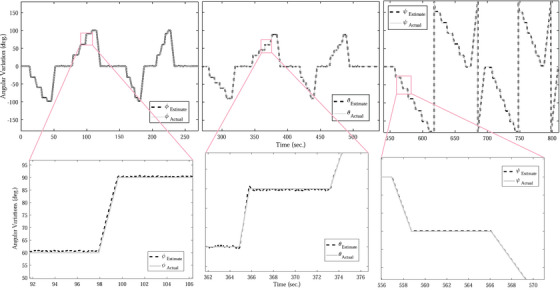
The angular variations for the developed system and a reference (computer numerical control [CNC] machine). The sensor is attached to CNC's end‐effector and both of them rotate at different angles from 0 to 810 s. In part one (from left to right), the end‐effector rotates around *x*‐ axis; in the second part, the end‐effector rotates around *y*‐axis; and the third part is related to the rotation end‐effector around *z*‐axis.

**FIGURE 4:**
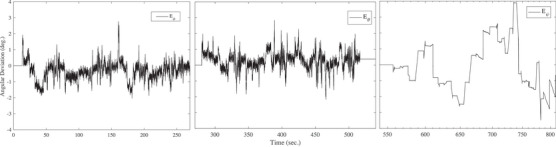
The angular deviation for the developed system from computer numerical control (CNC) machine. The errors are left to right, respectively, angular error rotation around *x*‐ axis, *y*‐axis, and *z*‐axis.

**FIGURE 5:**
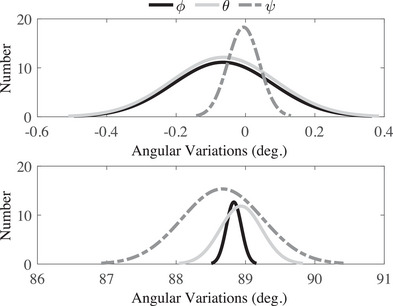
The outcome of the repeatability test for angles of 0 (top) and 90 (bottom) degrees for angles of ϕ, θ, and ψ. The *y*‐axis relates to the number of repetitive movements at 0 and 90 degrees. Also, the x‐axis is the angular variations of the developed system.

In order to measure the accuracy of the developed sensor, a CNC system is considered as reference and the sensor is rotated at different angles on a CNC system. To this end, the sensor is firmly attached to the CNC end effector and then rotated with various angles from 0 to ‐100 anf, then 0 to 100 in the direction of the *x*‐axis from 0 to 270 s. It is then rotated around the *y*‐axis from 270 to 540 s in various angles from 0 to ‐90 degrees and then 0 to 90 degrees and finally around the *z*‐axis from 0 to 180 degrees and then 0 to ‐180 for 540 to 810 s that all of the rotation is shown in Figure 3. It should be noted that when the sensor rotates around one axis, it does not have any rotation around the other two axes, and the sensor is fixed around those axes. The obtained results in Figure 4 illustrate the difference between the estimated angular variations and the actual value (CNC) that mean errors are ‐0.22, 0.19, and ‐0.15 as well as standard variation (SD) are 0.73, 0.71, 1.5, respectively, for ϕ, θ, and ψ angles. In order to obtain the accuracy of the developed system, the root mean square error is calculated by the following equation:

(16) $$RMSE=\sqrt{\frac{\sum_{j=1}^{m}{\left(A_{e,j}-A_{r,j}\right)}^{2}}{m}}$$

where *m* is the number of samples for the duration of 800 s in Figure 3. $A_{e,j}$ is defined as the estimated angular variation by the developed system and $A_{e,j}$ is the actual variation by the CNC that is considered as a reference. The obtained results show that the root mean square error of the proposed system for ϕ, θ, and ψ is 0.8, 1.07, and 1.56 degrees for static error and 1.01, 1.34, and 1.61 degrees for dynamic error that are less than result in [30]. In other words, the dynamic and static error is less than 3.6 degrees and 2.5 degrees in [30]. Thus, an improvement of 40% is obtained at least. When the developed sensor is attached to the trajectory of the CNC machine and both of them are constant, the error between the estimated angles of the developed system and the CNC machine is called static error. However, when the system and trajectory are turned, the difference in the angular variation between the developed sensor and reference is defined as dynamic error. In order to perform the repeatability test, two angles of 0 and 90 degrees are considered and the developed sensor is turned 50 times by the CNC from 0 to 90 degrees for angles of ϕ, θ, and ψ that the outcome is shown in Figure 5. The obtained result shows that the ψ has the best result than other angles while ϕ has a better result for 90 degrees because the normal distribution is more narrow.

### 4.2 Tests

**FIGURE 6:**
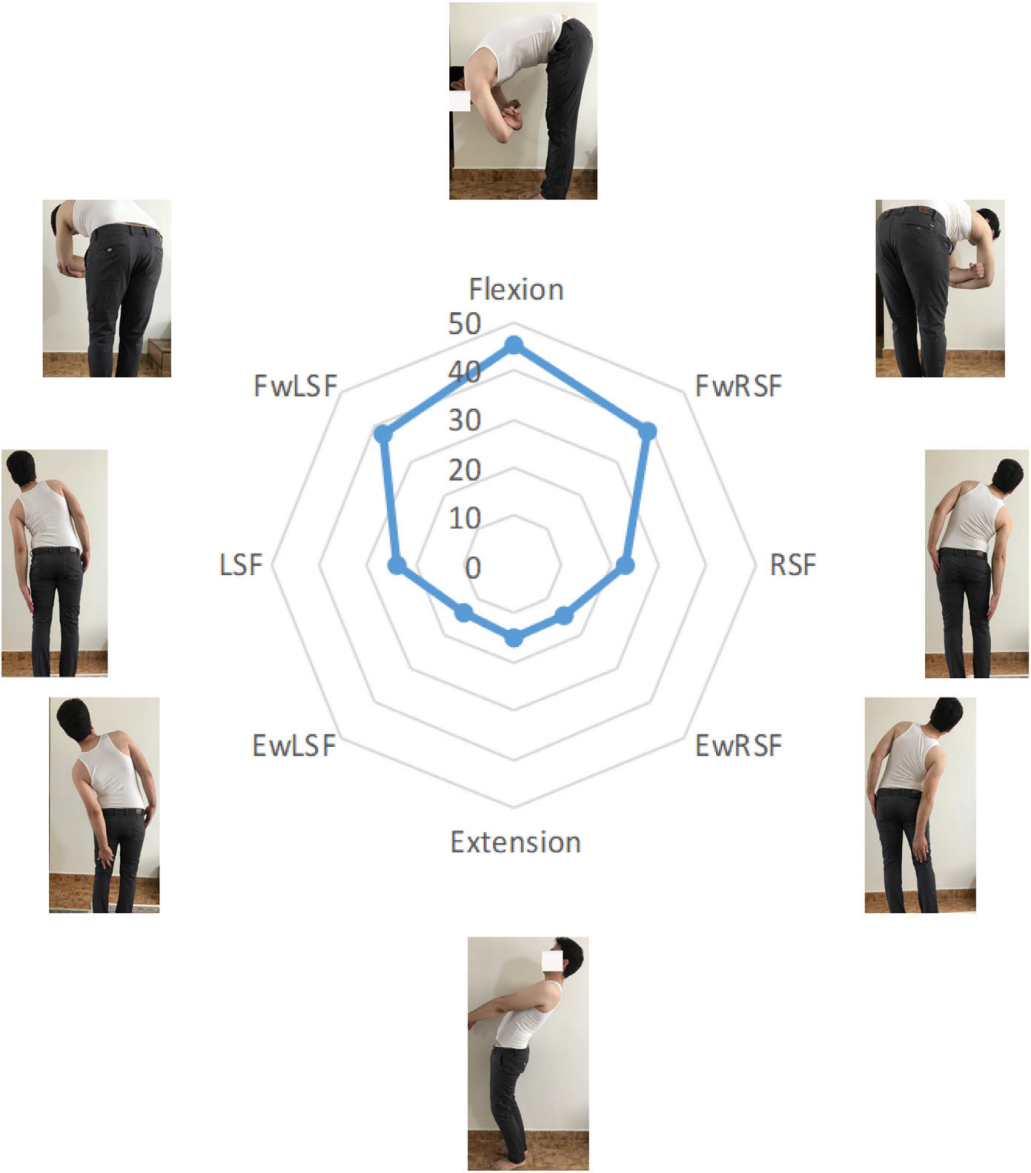
The obtained results (mean) for healthy volunteers' combined movement examination (CME) radial plot (in degrees) that consist of flexion with left side‐flexion (FwLSF), flexion with right side‐flexion (FwRSF), left side‐flexion (LSF), right side‐flexion (RSF), extension with left side‐flexion (EwLSF) and extension with right side‐flexion (EwRSF).

This system can be used for investigating the improvement in the spine lumbar. First, 10 healthy individuals (five male and five female) between the age of 31 and 38 years old are asked to incorporate in the test of Figure 6. It should be noted that a clinician approves the healthy persons with healthy spines for the tests as a reference that the obtained results for the test are shown in Table 1. All features of the reference volunteers are illustrated in Table 1. It should be noted that the obtained result in Table 1 is considered as the reference for the whole of the paper. Furthermore, 10 patients with ages 32–38 and BMI 21–28, who have problems in their lumbar, are asked to incorporate in the test. The patients are in similar situations with the reference (Table 2). Then, the volunteers are asked to perform the movements mentioned in Figure 6 that involve the eight CME positions by the examiner until their comfort limits. It should be noted that in a relaxed standing position, participants had their lumbar lordosis (angle between *L*_1_ and *S*_1_) that is considered zero starting position in the centre of the radial plot in Figure 6. Maximum data values for each CME position are recorded according to a pre‐defined sequence consisting of flexion, flexion with left side‐flexion (FwLSF), flexion with right side‐flexion (FwRSF), left side‐flexion (LSF), right side‐flexion (RSF), extension, extension with left side‐flexion (EwLSF) and extension with right side‐flexion (EwRSF).

**TABLE 1:** The angular variations of the lumbar spine of healthy male and female volunteers of specific ages and BMI are considered as reference.

Gen.	Age	*N*.	BMI	Flexion	FwRSF	RSF	EwRSF	Extension	EwLSF	LSF	FwLSF
Male	31–38	5	25.3(1.9)^1^	46.1(1.75)	39.2(1.61)	23.5(1.21)	12.9(1.06)	15.4(1.02)	12.7(1.09)	22.6(1.15)	38.5(1.51)
Female	31–38	5	22.8(2.3)	49.3(1.81)	38.2(1.41)	24.5(1.25)	13.5(1.02)	16.1(1.03)	13.7(1.01)	22.9(1.32)	40.5(1.53)

^1^ Mean (SD).

**TABLE 2:** The angular variations of the lumbar spine for male and female patients of specific ages and BMI before hand therapy, who incorporated in the test.

Gen.	Age	*N*.	BMI	Flexion	FwRSF	RSF	EwRSF	Extension	EwLSF	LSF	FwLSF
Male	32–38	5	25(2.23)^1^	49.7(2.87)	34.6(2.24)	22.2(2.6)	14.8(1.36)	13.8(4)	15.6(2.71)	22.9(2.36)	37.4(3.72)
Female	33–37	5	22.6(2.08)	42.1(0.71)	40.2(3)	18.4(3.23)	14.6(0.48)	16.2(2.4)	15.4(2.6)	23.8(2.95)	38.9(3.19)

^1^ Mean (SD).

For further investigation, two volunteers (Volunteer 1 and Volunteer 2 in Table 3) are considered, who suffer from problems in the spine lumbar. Both the volunteers were in very good health with no complaints of dominant psychosocial factors, trauma, or systemic disease. They had only experienced mild low back tightness 1–2 times per year. However, neither had experienced the same pain location or intensity as their presenting complaint. Indeed, the clinician performed some therapy for the patients in the first session and asked them to do some exercise and then the mentioned tests in the paper until the next session. The patients do the exercise using the developed system, and the result is compared to the reference in Table 1. After 7 days and doing the tests, the result is sent to the clinic, and if there is a high level of error between the tests and reference, they have to refer to the clinic for more hand therapy by the clinician.

**TABLE 3:** The angular variations of the spine lumbar of some volunteers after manual therapy and repeating the tests.

Vol.	Sex	Age	BMI	Therapy	A	Flexion	FwRSF	RSF	EwRSF	Extension	EwLSF	LSF	FwLSF	$E_{a}\left(\%\right)$
1	Male	32	22	After	25.2	45.1	38.6	22.9	12.2	14.5	13.1	21.7	37.2	3.5
				Before	20.1	48.2	37.1	19.5	14.2	10.3	17.4	20.2	40.7	15.5
2	Female	37	21	After	32.2	48.5	38.3	22.5	14.1	16.2	13.1	24.2	38.1	3.9
				Before	23.8	41.4	41.9	14.2	14.7	16.4	17.2	20.1	40.4	14.6
3	Female	36	20	After	30.8	49.2	38.5	21.9	14.3	16.1	14.8	21.5	40.2	4
				Before	38.6	42.1	39.3	21.8	14.3	17.1	15.5	22.3	43.8	8
4	Male	32	24	After	27.3	47.8	40.2	24.5	14.9	16.2	14	23.8	40.1	6.3
				Before	34.6	50.1	35.8	22.5	14.2	18.2	18.4	24.1	34.7	13.9
5	Female	33	23	After	29.5	48.9	38.6	23.2	11.8	16.2	12.9	21.7	39.6	4.2
				Before	22.2	43.1	42.5	19.6	14.2	18.7	11.5	24.3	36.5	12.2
6	Male	38	25	After	25.2	44.5	40.5	22.1	12.6	15.5	13.9	23.5	40.5	4.3
				Before	31.5	45.6	35.5	20.1	17.3	18.2	14.5	24.5	42.2	13.7
7	Female	37	24	After	34.2	48.5	38.3	22.5	14	16	13.1	24.2	38.1	3.8
				Before	28.8	42.5	35.4	15.8	14.5	16.5	18.2	28.1	37.2	16.3
8	Male	36	26	After	26.7	46.9	39.5	23.5	13.6	16.5	14.6	23.5	39.5	4.6
				Before	18.5	51.2	32.1	26.1	14.3	11.2	16.2	20.5	35	15.5
9	Male	34	28	After	24.6	45.3	39.1	25.1	13.7	15.9	14.1	24.2	38.1	4.7
				Before	21.2	53.1	32.3	22.5	14.3	11.2	11.5	25.2	34.5	13.3
10	Female	35	25	After	35.1	49.2	38.1	22.8	13.9	16.3	14.2	23.4	39.9	2.4
				Before	25.4	41.5	42.2	20.5	15.4	12.2	14.5	24.2	36.5	12.8

**FIGURE 7:**
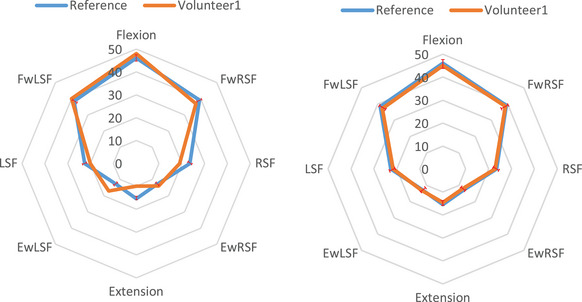
The obtained result for volunteer #1: (left side) before manual therapy and (right side) after manual therapy. The blue plot is the mean value of healthy male persons and the red dash is tolerance ranges for male volunteers that are extracted based on Tables 1 and 3. The brown is related to volunteer 1.

**FIGURE 8:**
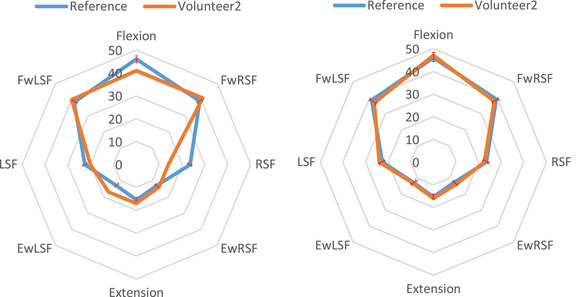
The obtained result for volunteer #2; (left side) before manual therapy and (right side) after manual therapy. The blue plot is the mean value of healthy female persons and the red dash is tolerance ranges for female volunteers that is extracted based on Table 1 and 3. The brown is related to volunteer 2.

**FIGURE A1:**
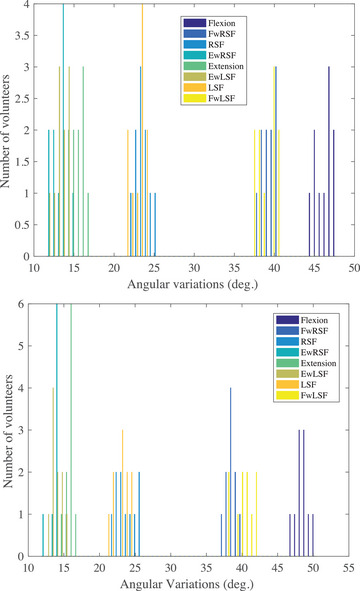
The normal distribution of all case studies is male (top) and female (bottom) healthy volunteers and also patients after hand therapy.

In volunteer #1, the most painful CME movement was a lumbar extension. In volunteer #2, the patient complained of right‐side LBP, and the most painful CME direction was right‐side flexion (RSF). Volunteer #1 is a 32‐year‐old man who complained of an acute exacerbation of central, constant lumbar stiffness and intermittent pain with movement. This case was attended for CME examination and manual therapy. The first result was the brown plot in Figure 7. After two tries on two different days, the obtained result is in Figure 7, right side $E_{a}$ decreases to 3.5% according to Equation (2). Case #2 is a 37‐year‐old woman who presented with an acute exacerbation of right‐sided, intermittent, mechanical LBP. This patient attended four sessions of manual therapy. As in the past case, at first, there is a meaningful difference between a healthy plot and the plot in Figure 8, left side. Then, after four sessions, the amount of $E_{a}$ decreases less than 3.9% (Figure 8, right). The difference between the plot of the healthy and unhealthy person is calculated by Equation (2), and Figures 7 and 8 illustrate the error decline after performing repetitive tests. The tests are performed for other patients, and the obtained results are recorded in Table 3. It should be noted that a numerical value is obtained to indicate the quality of the patient's movement by comparing the two plots. When the number goes to 0, the better quality is for the movement ($E_{a}$ in Table 3). Furthermore, the normal distribution of all case studies from healthy persons and patients after hand therapy is shown in Figure A1 in the Appendix. The obtained results reveal that angular variations of the eight positions are tolerated in an interval for the healthy volunteers and patients after hand therapy; this interval is shown in Figures 7 and 8 with a red dash. Thus, when patients are in the interval, their result is acceptable. Additionally, as Table 3 shows, the proposed system was tested on a specific group of male and female volunteers of ages 31–38 and 20<BMI<28. Moreover, the proposed system can measure the angular variations for other groups that are out of the range with different BMI and age. First, the correct references are chosen for every group and then patients who are in similar conditions with references are considered.

## 5 DISCUSSION

In this section, this developed system is compared with existing works from different aspects and discussed further. The first wearable system proposed in [31] was capable of measuring the position of an unobserved spine. Their system consisted of three electromagnetic inclinometers that measured the angle of inclination in the parabolic field. Also, their systems were attached to the chest, thighs, and legs. An analog‐to‐digital converter is included to transmit measured angles to 4‐bit digital signals that allow digital reconstruction from trunk, thigh, and leg angles. The system was suitable for classifying the situation as “poor” or “good.” However, there is no formal assessment of the accuracy of the system in [31]. Furthermore, an IMU‐based wearable system for office workers was developed in [32] that has been reported to be sensitive enough to report lumbar problems for users. Their study was only a preliminary recommendation and approval of the prototype, and studies on their validation in office staff are ongoing [32]. In another study, spinal movements were evaluated using Xsens sensors, and six volunteers were selected to perform the required tests [33]. However, the sensor used in their work is a very expensive sensor in the market, and its prices are from $1000 to $3000. Thus, it is not economical to use this sensor for home applications. In addition, the sensor system is not wireless and creates problems for the user. In another work, lumbar curve analysis was performed by five sensors [30]. However, there is no mention of the type and accuracy of the sensors, but they have extracted the curvature of the waist with this method.

The most important work related to this letter is in [11, 12, 13] which, with the help of a computer, identified the number of spinal deviations and provided solutions to eliminate them. In the three references, an expensive system based on the IMU is used to estimate the angular variation for the study of lumbar movements, which has disadvantages such as having bulky wires and requiring a computer. In addition, patients cannot use it at home and it is limited to the clinic. These problems can cause limitations when testing. For example, being wired during testing can greatly reduce the quality of work. However, the proposed system is wireless and lightweight, and every individual can be transferred easily. As mentioned earlier, a transceiver (NRF series) is used for sending and receiving data. The NRF has some advantages than Bluetooth and Wi‐Fi. The Bluetooth and Wi‐Fi benefit UART protocol, and the maximum rate for sending data is limited to 119,400 bits per second. However, NRF is connected to a microprocessor throughout the SPI protocol, and its bite rate is not limited to 119,400 and can be increased to 2 Mbs. Additionally, the existing system (Xsens) is very expensive at $699 while the proposed system is $50. Recently, there is another system in Xsens entitled Xsens Dots that had lower cost than the previous product in the market. Nevertheless, the price of the product is $140, which is more expensive than the work presented in this paper.

Another important feature of this system is less processing than the computer system in [11, 12, 13] that uses Kalman and expanded filters. Indeed, the developed system and also the proposed algorithm for obtaining the error between the estimated and reference is simple because the number of sample rates is similar in the reference and estimated angles, and it is recommended to use simple subtracting. In some movements, the sample rate is different and it needs to calculate the similarity between them. In this situation, a simple subtracting does not work and dynamic time warping can be used. However, the proposed method in the paper is based on simple subtracting because the amount of samples is the same. Therefore, the time processing for estimating the angle and also the improving quality of movement is reduced. Another feature of the proposed system is that the accuracy in the estimation of angular variations in comparison to the existing system is reasonable. As mentioned earlier, these systems use gyroscope and accelerometer sensors along with a compass sensor to measure. However, the absolute nature of gyroscopes raises the issue of bias error due to drift. Unlike accelerometers, which use the gravity vector as a reference, gyroscopes have no reference and are therefore unable to reset, thus leading to an accumulation of errors. This may be reduced by magnetic integration because these IMUs are calibrated by pointing to the earth's magnetic field. However, because magnetometers cannot distinguish between the earth and other magnetic fields, they may be prone to interference with hard iron distortion [34]. As a result, public IMUs contain a combination of all three monitors on all three axes θ, ϕ, and ψ [32]. In this developed system, the sensor data were combined and the error rate was greatly reduced to less than 1.61 degrees for dynamic and static error.

By developing a very small, inexpensive portable sensor, patients can quickly extract their spinal motion analysis and try to improve the existing problem in the lumbar spine. As a future work, the system can be improved in hardware and combined with the internet of things to send the data to a server for further processing and have a large data set that helps the researcher estimate the behaviour of the lumbar spine using machine learning and artificial intelligence.

## 6 CONCLUSION

Here, a system of miniature, inexpensive, and portable based on an accelerometer, gyroscope, and compass motion sensors was developed to study spinal movements. To do this, first, the angular variation of movement was estimated using a complementary filter. Then, the obtained result showed that the accuracy of the developed system is higher than similar works. The developed system is mounted on the lumbar spine of volunteers who performed several defined movements, and their data were compared to some healthy persons as a reference. After manual therapy and repeating the exercise, their problems improved over time and the obtained result illustrated the claim. Thus, patients can improve the problem in their lumbar spine by using the developed system.

## AUTHOR CONTRIBUTIONS

**Reza Abbasi‐Kesbi**: Conceptualization; investigation; software; supervision; validation; visualization; writing—original draft; writing—review and editing. **Mohammad Fathi**: Conceptualization; data curation; formal analysis; methodology; resources. **Seyed Zaniyar Sajadi**: Conceptualization; formal analysis; investigation.

## CONFLICT OF INTEREST STATEMENT

The authors declare no conflicts of interest.

## Data Availability

The data that support the findings of this study are available from the corresponding author upon reasonable request.

## 1

In Figure A1, the normal distribution of angular variation of all case studies of males and females is shown to consist of healthy volunteers and also patients after hand therapy.

## A.1 Instructions for patients

First, the patients are asked to move within their comfortable limits and then they perform the eight CME positions. The patients do the test in the clinic when the clinicians supervise their work. After that, they repeat the test several times at home to improve their movement, and their results are sent to the clinician. Whenever the patients have pain in their body, they are never permitted to increase the limitation of the movement in the eight CME positions and they have to call to the clinician for more guidance. If patients need more hand therapy, they have to be referred to a clinic. Otherwise, they can continue their test until reach a reasonable result.
